# Poisoning by Strychnine

**Published:** 1879-09

**Authors:** W. H. Smith

**Affiliations:** Shell Rock, Iowa


### ARTICLE XI.

*Poisoning by Strychnine — Prompt Recovery after Administration of Potassi. Bromid.*

On the evening of July 7th, 1879, I was hastily summoned to a livery office, where the young man in charge, aged about twenty, was affected with very severe convulsions. I could elicit nothing further than the answer "No" to all questions, and the surroundings gave no clue to the mystery. I lost no time in giving an heroic emetic, with orders to those in attendance to hurriedly get some milk and warm water, while I returned to my office — a distance of one block — for medicine. While gone, his employer succeeded in ascertaining that strychnine had been taken. I at once gave milk and 4 grams of the bromide of potassium, waiting sufficiently long for the effects of the emetic, with no results. Of the bromide, gave from 15 to 30 grams within one hour, during the last half of which the rigidity became less and less marked, and was soon followed by vomiting and nearly complete relaxation.

With occasional muscular twitchings, the patient fell asleep, and awoke to recovery without an unfavorable symptom, resuming his work again on the 9th.

Since his recovery he has stated that the day before his illness he purchased five cents worth of strychnine, the whole of which was taken, making a piece, as he says, about the size of a pea. This was taken about six o'clock in the evening of the 7th, and was soon followed by muscular twitching, which continued. When found, he was in a continuous and persistent spasm from head to foot, with an alarming opisthotonos, and extremities cold to body. For awhile it was difficult to administer medicine, but, keeping the jaws apart by the introduction of a fan-handle, small amounts were constantly introduced. The point of special interest to me is the promptness with which the patient was relieved by the rather heroic use of bromide of potassium.

W. H. SMITH, M.D.

SHELL ROCK, IOWA, July 11th, 1879.

---

A LIVELY CITY. — The editor of the *Louisville Medical News* says they have in that city, now, five medical journals, and four medical colleges, and intimates that there are yet from ten to twenty members of the profession who are still without professorships. It is evident that there is both room and material for one more medical school in Louisville.

